# Older Adults and Digital Society: Scientific Coverage

**DOI:** 10.3390/ijerph16112010

**Published:** 2019-06-05

**Authors:** José Álvarez-García, Amador Durán-Sánchez, María de la Cruz del Río-Rama, Ronny Correa-Quezada

**Affiliations:** 1 Financial Economy and Accounting Department, Faculty of Business, Finance and Tourism, University of Extremadura, 10071 Caceres, Spain; pepealvarez@unex.es (J.Á.-G.); amduransan@unex.es (A.D.-S.); 2 Business Organisation and Marketing Department, Faculty of Business Administration and Tourism, University of Vigo, 32004 Ourense, Spain; 3 Department of Economics, Universidad Técnica Particular de Loja (UTPL), 11-01-608 Loja, Ecuador; rfcorrea@utpl.edu.ec

**Keywords:** older adults, information and communication technologies, ICTs, bibliometric Study, WoS, Scopus, scientific coverage

## Abstract

While there is a progressive ageing of the population, we are witnessing a rapid development of new information and communication technologies (ICTs). Although for most of society this technology is within reach, there are population segments for whom access is limited, especially adults who are considered of old age. Due to the relevance that the relationship between ICTs and older adults acquires in today’s society, it is necessary to carry out an analysis of the scientific literature in order to understand the knowledge structure of this field. In this research, a comparative bibliometric analysis of 172 documents published in the Web of Science (WoS) and Scopus databases was carried out until 2018 and is complemented by a co-citation analysis. The results show that this subject is incipient and is in its exponential growth stage, with two thirds of the production concentrated in the 2012–2018 period. Four out of five authors are transient with a single authorship and the collaboration level is high. The most productive country is Germany followed by the United States and Australia.

## 1. Introduction

The ageing of the world population and the increase in life expectancy is one of the main achievements of modern societies. Nowadays, people can aspire to live more than 60 years, mainly thanks to scientific advances in medicine, nutrition, and technology. However, ageing also poses great challenges and issues in the 21st century that must be dealt with. In 2017, it was estimated that there were more than 962 million people over 60 years of age on the planet, representing 13% of the world’s population and a growth rate of around 3% per year. Furthermore, it is expected that the number of people in this age group will have doubled by 2050, with more than 2.1 billion people, and will have tripled by 2100, reaching 3.1 billion [1].

At the same time, as there is a progressive ageing of the population, we are witnessing the rapid development of new information and communication technologies (ICTs). Both changes, increased life expectancy and advances in information technology, seem to be unstoppable and their implications unpredictable. Due to the transformative effect of ICTs in all areas of society, there are many researchers who agree on noting that the ability to use them is an essential precondition for a good performance of daily life activities in the so-called information era, so their universal access should be guaranteed and the disparities between the different groups that use them should be reduced [2]. Although for the majority of society, new technology is within reach, there are population segments whose access is restricted or limited.

In this regard, and with the aim of overcoming the generational digital gap, older adults deserve special attention since they are the population group that adapts more slowly to the rapid changes that have taken place in the digital society [3]. For a real integration of the elderly to take place in our increasingly technocratic societies, the implementation of ICTs in their lives should be considered a useful instrument with which they interact and connect with the outside world [4], and therefore, promote active ageing and achieve a higher quality of life [5].

Given the relevance that the relationship between ICTs and the elderly has been acquiring within the academic world, it is necessary to carry out an in-depth analysis of the extensive and fragmented related academic literature. Thus, in this article a bibliometric review is carried out with the objective of obtaining a complete vision of this research area and its current state. With this objective, the bibliographic records of scientific articles published in the Web of Science (WoS) and Scopus databases until 2018 were reviewed. The overlap and singularity between both databases were also measured. The novelty of this study is that there is no work with similar characteristics in this field of knowledge.

The article is structured as follows. After the introduction where the subject is contextualized and the objective is proposed, in the second section, a brief review of the literature is collected in order to establish the theoretical framework (ICTs and older adults). The methodology is then described, and in Section 4, the results obtained are shown and discussed. Finally, in Section 5, the main conclusions and limitations associated with the investigation are presented.

## 2. Theoretical Framework 

Demographic ageing is a trend established in most countries of the world [6], and usually coincides with the retirement age, between 60 and 65 years in developed countries, whereas in developing countries it begins the moment an active contribution to society is no longer possible [7]. Due to these discrepancies, the World Health Organization defined the concept of ageing as the decrease in the rate of children and young people with respect to the increase in the rate of older people (+60 years) [8].

In contrast to this ageing population, adaptation to new information and communication technologies (ICT), defined as devices, tools, equipment, and electronic components capable of manipulating information [9], by older adults takes place at a lower rate compared to other age groups, resulting in an age-based digital gap [10]. However, the use of ICTs also differs within this segment, decreasing their use as age increases [11]. Furthermore, if it considered that older adults are already susceptible to socioeconomic inequalities [12] and that more and more public services are only provided online, non-access and the lack of digital literacy can contribute to greater inequality related with age [13].

Although studies that point to age as the main curb to the use of ICTs by older adults can be found in the academic literature [14], there are other factors that play a crucial role in the use (enhancers) or non-use (barriers) of ICTs. According to Neves et al. [13], these factors are classified into three groups: attitude, functional, and physical. In terms of attitude reasons, these are on the one hand the lack of confidence in their ability to deal with ICTs [15], and on the other hand, the lack of interest and need as a result of the false belief that ICTs are not appropriate for this age segment due to their difficulty and usefulness [16]. These reasons may explain the lack of adoption or the low use of information and communication technologies by older adults. With regard to functional reasons, their lack of access, sometimes caused by the economic situation [17], or the lack of digital literacy are also among the most recognized reasons for not using ICTs [18]. Finally, in terms of physical reasons, physical conditions can affect positively or negatively. For example, if the problem is visual or related to arthritis, the use of ICTs will be negatively affected [19]. On the contrary, users with increased mobility problems can spend more time managing ICTs by spending more time at home [20].

Similarly, other authors such as Morrell et al. [21] found that some of the main barriers for older people not using information technologies are the lack of access and knowledge and sometimes, their high cost. For Wang et al. [22], four factors influence the acceptance of information technology by older adults (enhancers), in this order: satisfaction of needs, availability, perceived usefulness, and public acceptance.

In general, the academic literature suggests that the adoption of technology is a complex issue and it is influenced by a great diversity of sociodemographic factors, attitudes, and cognitive abilities, with complex relationships between these variables [2]. Therefore, it seems clear that older adults are not technophobic and are willing to use ICTs competently [23]. However, they do not trust their ability to use these systems successfully [24], despite having cognitive abilities such as memory and the processing speed necessary for the successful performance of tasks based on ICTs [25].

In this context, in which the adoption of technology is a complex issue, there are investigations that seek to identify the factors that influence the acceptance of technology by users and especially by the group of older adults [3,23,26,27,28] (see in Ma et al. [29] the determinants of the acceptance of ICTs innovations by older adults). So, the models of acceptance of the technology arise. The Senior Technology Acceptance Model (STAM) is a model that explains the acceptance of the technology in older adults and is one of the few technology acceptance models that focused on older adults and general gerontechnology. STAM was proposed by Renaud and van Biljon [30] in order to consider the unique characteristics, capabilities, and limitations of older people regarding technological acceptance. This model is a variant of the Technology Acceptance Model (TAM) model, proposed by Davis et al. [31] with the aim of explaining the behaviors that push consumers to accept technologies. In the TAM model it is suggested that for users, when faced with a new technology, there are a set of factors that influence their use decision: perceived usefulness (PU) defined as the degree to which a person believes that using a particular system would enhance his or her job performance [31], reported ease of use (PEOU), the degree to which a person believes that using a particular system would be free from effort [31], perceived enjoyment, degree in which a person finds a pleasant activity when using technology [31]. The Senior Technology Acceptance Model (STAM) model adds age-related health and ability characteristics of older people who, according to some studies, are better predictors than the conventionally used attitudinal factors (usefulness and ease of use) [32,33]. This model was also adapted by Smith [34] applying the same to e-commerce in the case of older adults.

On the other hand, many authors have highlighted both the socioeconomic and health benefits derived from a greater access and use of ICTs: they help to reduce social isolation among older adults [14,35], they facilitate daily life in basic activities such as shopping or management, thereby increasing their quality of life and favoring active ageing [20], reduces the probability of a depression categorization [36] or they decrease perceived life stress [37]. However, as Aroldi et al. [38] point out, it is not possible to quantify exactly if the adoption of technologies guarantees the elderly´s inclusion and participation, making it necessary to investigate further before being able to fully understand the role played by technologies in active ageing, especially in the domestic environment [39].

However, without any doubt, the greatest benefits obtained by the elderly from the use of ICTs are shown in everything related to their health and care. For a long time, new and innovative approaches based on technology have been emerging to support the care of the elderly. As C.E. Koop revealed in 1995, cutting-edge technology, especially related to communication, will allow for the greatest advances in public health. Communication technology can provide each household with access to health information 24 hours a day, 7 days a week, promoting well-being and prevention [40]. “Assistive technology,” understood as technological innovations that help improve the care of the elderly or disabled [41], can, in certain cases, replace, or at least complement, their personal assistance [42]. Although they are commonly accepted [43], on some occasions, users have expressed concern about their difficulty of use, lack of human contact, the need for specialized training [44] and privacy [45].

The incorporation of ICTs into the home and the consequent automation of care for the elderly enables them to live independently and safely in a family environment, significantly reducing the costs of medical care thanks to the early detection of a health problem, even in remote places. For Weiner et al. [46], ICTs are the structural component that most influences the improvement of the process of providing medical assistance, which leads to higher health levels and, therefore, increases the functional independence of the elderly.

## 3. Methodology 

Bibliometrics is a widely used method to analyze specific areas of research and draw valuable conclusions [47], using objective information that is easy to manage [48], with the aim of facilitating decision-making and channeling the researcher’s efforts [49]. Thus, bibliometrics is considered an interdisciplinary science focused on the quantitative analysis of bibliographic data through statistical and mathematical tools [50]. On the other hand, the publication of articles in scientific journals is one of the most used mechanisms for disseminating research results and as a whole, it constitutes a representative sample of international scientific activity [51].

Thus, the systematic search of the bibliography related to a field of study is the first step in all research, allowing to establish its theoretical framework as well as to set the hypotheses that will lead the way for the study. Therefore, it is essential for this initial stage to be carried out in a structured and non-random way, and the use of bibliometric methods is necessary at this point.

**Figure 1:**
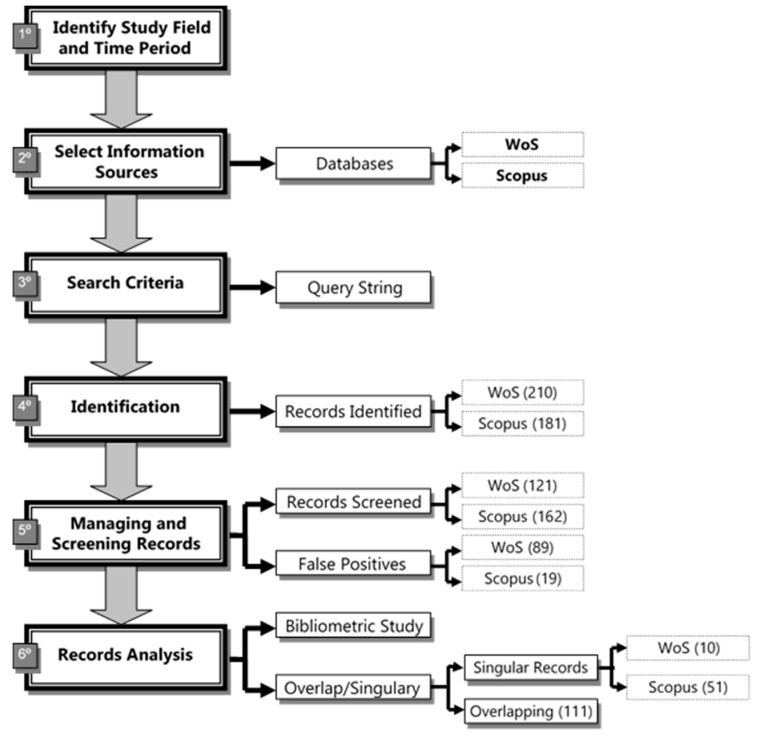
Methodological scheme of the bibliometric analysis. Source: own elaboration.

Following Rowley and Slack [52], who propose to design a mental map in order to establish the steps to follow in the process of systematic search of bibliography, in this work the following structure is followed (Figure 1).

Bibliographic databases are defined as digital collections of references to published sources, in particular to journal articles [53]. They become an essential resource for any bibliometric study, enabling to analyze the scientific activity carried out by researchers, centers, regions, and countries.

The existence today of a multitude of national and international databases, both generic and specialized, makes it necessary to evaluate which of them makes a greater coverage of the area to be studied, since the choice adequacy will largely depend on the validity of the results obtained [54]. In this research, the Web of Science (WoS) and Scopus databases were chosen, which are both worldwide references that have been subject to comparisons from the perspective of their coverage: collected articles, journal titles, thematic and geographical areas, affiliation, languages, citation analysis [55,56].

In this paper, the search for terms was chosen in order to track documents (January 2019), a strategy that allows for tracking classified journals within all thematic areas, being, therefore, more exhaustive [57]. Query string (year of publication 2019) for the subject Older Adults and Information and Communication Technologies (ICTs) is:

WoS: (TI = (“old * adult *” OR “silver surfer *” OR “old * population” OR “old * people” OR “third age” OR aged OR “old * person *” OR elder * OR ageing OR aging) AND TI = (“information technolog *” OR “communication technolog *” OR ict *)) AND LANGUAGE: (English) AND TYPES OF DOCUMENTS: (Article) and (Review)

Scopus: (TITLE (“old * adult *” OR “silver surfer *” OR “old * population” OR “old * people” OR “third age” OR aged OR “old * person *” OR elder * OR ageing OR aging) AND TITLE (“information technolog *” OR “communication technolog *” OR ict *)) AND DOCTYPE (ar OR re) AND PUBYEAR < 2019 AND (LIMIT-TO (LANGUAGE, “English”))

In this research, only articles and reviews published in scientific journals considered quality references contrasted by a blind peer evaluation process were selected. The final result of the search was 121 articles published in WoS and 162 articles in Scopus. A database was constructed in the Microsoft Excel software program for the calculation of bibliometric indicators.

## 4. Results

### 4.1. Production

The temporal distribution of the selected articles (Table 1), shows that the first work dates from 1990 (Scopus) and it is not until 2012 when there is a real interest in this subject by the scientific community. A total of 46.76% of WoS articles and 38.89% of Scopus articles are published in the 2015–2017 period, which makes it possible to deduce that it is a current field of study. The low production in 2018 can be explained by the fact that at the time of the search, January 2019, many of the papers completed in the last months of 2018 had not yet been indexed.

**Figure 2:**
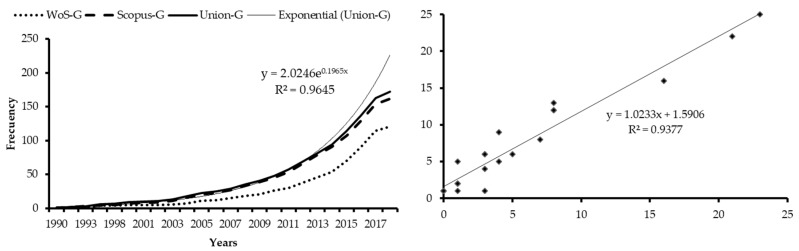
Production: Growth and Correlation for years. Source: own elaboration.

After an initial period with specific publications, called Precursors (law of exponential growth of Price, [58]), as of 2012 there is a turning point in the growth curve of the production of papers on the elderly and ICTs and a second stage of Exponential Growth begins. Figure 2 shows that it is foreseeable that this behavior will be maintained in the next few years before moving on to the last phase of Linear Growth, where the contribution of publications in this field, mostly reviews, will decrease. There is a strong correlation between the number of articles indexed per year in WoS and Scopus with R^2^ = 0.9377.

### 4.2. Citations

The documents indexed in WoS (121) received 1557 citations (12.9 citations/document), with an h-index of 20 (20 papers obtained 20 citations or more). With respect to Scopus, its 162 indexed articles obtained 2940 citations with an average of 18.1 citations/article and an h-index of 26. In WoS, the year with the highest number of citations (208) was 2009, while in Scopus it was 2004, with 360 citations.

**Figure 3:**
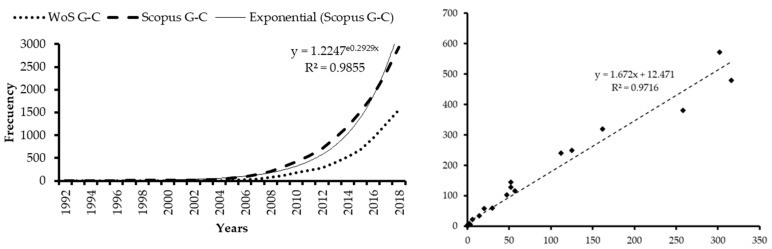
Citations: Growth and Correlation citations received for years. Source: own elaboration.

On the other hand, there is a constant growth in the number of citations that the articles receive annually (Figure 3), which has constantly been occurring since 2007, reaching the maximum number of 302 citations in WoS and 572 citations in Scopus in 2018. There is also a strong correlation between the number of citations received per year between both bases, with R^2^ = 0.9716. A more detailed citation analysis shows that only 2.48% (three) of WoS articles and 3.86% (five) of Scopus articles receive more than 100 citations, 4.96% (six) and 5.56% (nine) respectively, between 50 and 100 citations, 27.27% (33) and 30.25% (49) between 10 and 49 and 47.93% (58) and 49.38% (80) between one and nine. Only 17.36% (21) of WoS articles and 11.73% (19) of Scopus articles do not receive any citations. Note what was stated by Merigó et al. [59], articles published within the last 10 years may not have reached their maximum citation level yet.

According to the classification of articles based on their number of citations (Table 2), only three articles obtained more than 100 citations in both databases; in the first place, the paper by Selwyn [60], with 175 citations in WoS and 231 citations in Scopus, in the second place, de Charness and Boot [61] with 127 citations and 171 citations and in the third place, Heart and Kalderon [62], with 112 and 159 respectively.

On the other hand, there are articles that occupy a prominent position in the Scopus ranking, which are not indexed in WoS. This is the case of the article by Selwyn et al. [2], which leads the ranking with 293 citations or Magnusson et al. [68], which is in the 5th position with 101 citations.

### 4.3. Overlap and Singularity

A total of 111 articles of the 172 articles identified are overlapping (indexed in both databases), which represents 91.74% of WoS documents and 68.52% of Scopus documents. The remaining articles, 10 (8.26%) and 51 (31.48%) respectively, only appear in one of them. In the case of journals, the overlap percentage is 92.31% in WoS and 70% in Scopus. There are 7.69% and 30% single documents respectively.

On the other hand, the most common way to measure the degree of overlap between bases is by using traditional overlap (TO) of Gluck [70]. The higher the TO value, the higher the similarity degree is between the bases. The results indicate that there is a 64.43% similarity or seen otherwise, there is a 23.34% disparity between both bases.

(1) $$\%\mathrm{TO}\;=\;100\times \;\left(\frac{\left|WoS\;\cap Scopus\right|}{\left|WoS\;\cap Scopus\right|}\right)\;=\;64.43\%.$$

To know the percentage coverage of WoS with respect to Scopus and vice versa, relative overlap is used [71]:

(2) $$\%\mathrm{RO}\;\mathrm{WoS}\;=\;100\times \;\left(\frac{\left|WoS\;\cap Scopus\right|}{WoS}\right)\;=\;91.74\%.$$

That is, Scopus overlaps 91.74% of WoS articles. The % RO Scopus = 68.52%, that is, WoS covers Scopus 23.22% less than Scopus covers WoS.

The overlap differences may be due to the different indexing policies, but mainly due to the difference in the number of journals indexed between WoS and Scopus. Another important aspect to take into account is the relative singularity index of WoS and Scopus [72], which in addition to including the degree of overlap takes into account the percentage of single documents present in each of the databases. This index (Σsources × weight/total sources) enables to compare the coverage on a given subject. The higher the index value, the higher the singularity of the database is. Singularity is greater in Scopus with 31.48% of articles (8.26% in WoS) and 30% of single journals (7.69% WoS) and a Meyer’s index in the articles of 0.66 and 0.54, respectively, and 0.65 and 0.54 in the journals.

### 4.4. Authors

E. Hanson leads the ranking of the most productive authors (Table 3), with nine published papers. According to the criteria proposed by Lotka [73], there are no authors considered large producers, that is, with 10 or more publications. A total of 16.98% (99) of them are intermediate producers (between two and nine authors), while 484 (83.02%) are transient authors with a single authorship. Consequently, the Productivity Index is 1.21.

A Collaboration Index of 4.10 together with a collaboration level, ratio between the number of collaborative papers and the total number of papers, of (83.72%) shows a clear picture of the researchers’ collaboration level. None of the authors included in the most cited authors ranking has an individual authorship paper. The transience index is 83.01%.

**Figure 4:**
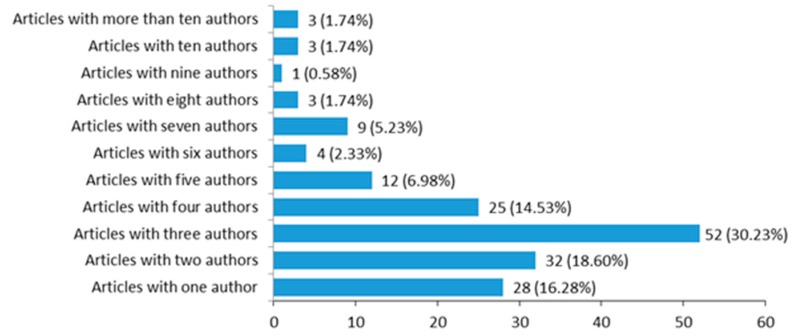
Collaboration. Source: own elaboration.

The highest percentage of articles, 30.23% (52) are signed by three authors, followed by 18.60% (32), which are signed by two authors and 16.28% (28) by one author (Figure 4). The existence of articles with more than 10 authorships can distort the previously seen collaboration index in a certain way. This is the case, for example, of The Lower Saxony research network design of environments for aging: towards interdisciplinary research on information and communication technologies in aging societies [74], with 75 signatures or information and communication technologies for promoting and sustaining quality of life, health and self-sufficiency in aging societies-outcomes of the Lower Saxony Research Network Design of Environments for Aging (GAL) [75] with 61 signatures.

By countries (Table 4), and according to the number of authors and authorships, Germany stands out with 18.38% (109) of the authors affiliated to some of its centers and 23.94% (169) of authorships, followed by the United States with 15.09% (88) and Australia with 8.06% (47). However, the United States is the country to which the databases attribute a higher affiliation of articles, 17.4% (21) in WoS and 16.60% (26) in Scopus. This country also receives the highest number of citations, in WoS 455, but not in Scopus, since with 26 articles, the same as the United States, the United Kingdom obtains 758 citations.

### 4.5. Journals

It is of great interest for researchers to know the most productive journals in their area of research. According to the law of Bradford [76], there is a small number of journals (Bradford’s Core) in each field that group most of the articles published related to that field. By calculating the so-called Minimum Bradford Zone (MBZ), number of articles equal to half the number of journals that produce a single article (108), and the ranking of journals arranged in descending order of productivity (Table 5), the Bradford Core is made up of those journals whose sum of articles was equal to the MBZ (54). This core is not well defined since there are 14 journals that compose it, nine of which only have three or two publications. Educational Gerontology stands out from the rest, with 11 articles followed by International Journal of Medical Informatics with six articles and Health Informatics Journal with five articles.

It is difficult to compare WoS and Scopus regarding the thematic areas in which journals are classified, where articles are included, since there is no clear correspondence in the denomination and content between both bases (Table 6). Despite this fact, certain similarities are found. Both in WoS and Scopus, most of the articles are integrated within health-related categories, Geriatrics and Gerontology (35) in the first one and Medicine (82) in the second one, and they are also the ones that receive the highest number of citations (563 and 2024). As expected, Computer Science occupies a prominent position (third place), with 19 articles in WoS and 33 articles in Scopus.

## 5. Conclusions

It is from the year 2012 when the interest in the subject of older adults and ICTs was aroused in the scientific community, therefore, it is a young subject. The growth in the production of articles has been constant since that year, concentrating two thirds of the total production in the 2012–2018 period. It is foreseeable that this behavior will continue in the coming years. With respect to the growth in the number of citations that publications receive per year, it is constant reaching its highest level in 2018.

There is no author considered a large producer (10 or more articles) and four out of five authors are transient authors with a single authorship, with the Productivity Index close to 1. E. Hanson leads the ranking of the most productive authors. A high collaboration level of researchers in this subject is observed; (Collaboration Index higher than 4 and collaboration level close to 85%).

By countries, considering the number of authors and authorships, Germany stands out followed by the United States and Australia. However, the United States is the country with the highest article indexing and the highest number of citations in WoS, but not in Scopus, since in this base it is the United Kingdom. The varied affiliation of researchers also demonstrates the enormous interest that the object of study arouses worldwide.

Finally, there is no well-defined core of journals, which collects most of the published papers. Educational Gerontology, followed by far, by the International Journal of Medical Informatics and the Health Informatics Journal is the one that publishes the highest number of articles. With respect to the subject areas in which journals are classified, where articles are included, there is no clear correspondence in the denomination and content between both bases, making comparison difficult. Despite this fact, certain similarities are found. Both in WoS and Scopus, most of the articles are integrated within categories related to health, Geriatrics and Gerontology in the first one and Medicine in the second one, and at the same time, they receive the largest number of citations. As expected, given that the study analyzes ICTs in relation to older adults, Computer Science occupies a prominent position (third place) in the ranking of thematic areas that contain the most articles.

The analysis and comparison of the two databases (WoS and Scopus), in order to determine which one is most influential in this field of study, due to its coverage, confirms that Scopus obtains the largest number of citations and collects a greater number of documents (almost one third of single documents and overlaps nine out of 10 of WoS articles).

Finally, it is important to consider the limitations of this research; the choice of databases and, on the other hand, the bias implied by the use of a specific search equation. As a possible future line of research, it would be interesting to extend the comparative study to other bases, expand the search terms including specific terms of the ICTs (internet, social networks, smart phones, etc.), perform collaborative analysis or deepen the content of the documents (bibliographic analysis).

## Figures and Tables

**Table 1:** Production of articles in Web of Science (WoS) and Scopus.

Year	WoS							Scopus						
	fi	hi	Fi	C	G-C	$\bar{X}$	h	fi	hi	Fi	C	G-C	$\bar{X}$	h
1990	0	0.0%	0	0	0	0.0	0	1	0.6%	1	22	22	22.0	1
1992	0	0.0%	0	0	0	0.0	0	1	0.6%	2	3	25	3.0	1
1993	1	0.8%	1	1	1	1.0	1	1	0.6%	3	4	29	4.0	1
1997	3	2.5%	4	9	10	3.0	2	1	0.6%	4	8	37	8.0	1
1998	0	0.0%	4	0	10	0.0	0	1	0.6%	5	0	37	0.0	0
2000	1	0.8%	5	64	74	64.0	1	2	1.2%	7	171	208	85.5	2
2001	0	0.0%	5	0	74	0.0	0	1	0.6%	8	0	208	0.0	0
2002	0	0.0%	5	0	74	0.0	0	1	0.6%	9	16	224	16.0	1
2003	1	0.8%	6	51	125	51.0	1	2	1.2%	11	351	575	175.5	2
2004	1	0.8%	7	173	298	173.0	1	5	3.1%	16	360	935	72.0	5
2005	4	3.3%	11	77	375	19.3	3	5	3.1%	21	127	1062	25.4	5
2006	1	0.8%	12	37	412	37.0	1	2	1.2%	23	62	1124	31.0	2
2007	3	2.5%	15	77	489	25.7	3	4	2.5%	27	82	1206	20.5	3
2008	3	2.5%	18	68	557	22.7	3	6	3.7%	33	189	1395	31.5	5
2009	3	2.5%	21	208	765	69.3	3	6	3.7%	39	306	1701	51.0	4
2010	5	4.1%	26	21	786	4.2	2	6	3.7%	45	30	1731	5.0	3
2011	4	3.3%	30	88	874	22.0	4	9	5.6%	54	145	1876	16.1	5
2012	9	7.4%	39	111	985	12.3	6	12	7.4%	66	163	2039	13.6	7
2013	8	6.6%	47	181	1166	22.6	5	13	8.0%	79	294	2333	22.6	7
2014	8	6.6%	55	117	1283	14.6	6	12	7.4%	91	178	2511	14.8	7
2015	15	12.4%	70	133	1416	8.9	7	16	9.9%	107	203	2714	12.7	9
2016	21	17.4%	91	94	1510	4.5	4	22	13.6%	129	133	2847	6.0	5
2017	23	19.0%	114	46	1556	2.0	4	25	15.4%	154	87	2934	3.5	5
2018	7	5.8%	121	1	1557	0.1	1	8	4.9%	162	6	2940	0.8	1
Total	121	100%		1557		12.9		162	100%		2940		18.1	

fi—frequency (number of articles published; hi—relative frequency; C—the total number of citations per year; G-C—total number of citations received for published articles; $\bar{x}$—Average; h—Hirsch’s index (the index h measures the number of “X of documents” that have received “X citations” or more, and at the same time does not have “X + 1 documents” with “X + 1 citations” or more).

**Table 2:** Ranking of the most cited articles.

Author/s	Age	Title	WoS			Scopus		
			R	C	C/Age	R	C	C/Age
Selwyn, N. [60]	14	The information aged: A qualitative study of older adults’ use of information and communications technology	1	175	12.5	2	231	16.5
Charness, N.; Boot, W.R. [61]	9	Aging and Information Technology Use: Potential and Barriers	2	127	14.1	3	171	19.0
Heart, T.; Kalderon, E. [62]	5	Older adults: Are they ready to adopt health-related information and communication technologies (ICT)?	3	112	22.4	4	159	31.8
White, J.; Weatherall, A. [63]	5	A grounded theory analysis of older adults and information technology	4	65	3.6	9	83	4.6
Hernández, E.; Pousada, M.; Gómez, B. [64]	9	ICT and Older People: Beyond Usability	5	63	7.0	8	83	9.2
Choi, N. [65]	7	Relationship Between Health Service Use and Health Information Technology Use Among Older Adults: Analysis of the US National Health Interview Survey	6	56	8.0	12	60	8.6
Vroman, K.; Arthanat, S.; Lysack, C. [66]	3	“Who over 65 is online?” Older adults’ dispositions toward information communication technology	7	54	18.0	10	66	22.0
Weiner, M.; Callahan, C.M.; Tierney, W.M.; Overhage, J.M.; Manlin, B.; Dexter, P.R.; McDonald, C.J. [46]	15	Using information technology to improve the health care of older adults	8	52	3.5	13	59	3.9
Fischer, S.H.; David, D.; Crotty, B.H.; Dierks, M.; Safran, C. [41]	4	Acceptance and use of health information technology by community-dwelling elders	9	49	12.3	11	60	15.0
Torps, S.; Hanson, E.; Hauge, S.; Ulstein, I.; Magnusson, L. [67]	10	A pilot study of how information and communication technology may contribute to health promotion among elderly spousal carers in Norway	10	49	4.9	15	48	4.8
Selwyn, N.; Gorard, S.; Furlog, J.; Madden, L. [2]	15	Older adults’ use of information and communications technology in everyday life	-	-	-	1	293	19.5
Magnusson, L.; Hanson, E.; Gorg, M. [68]	14	A literature review study of information and communication technology as a support for frail older people living at home and their family carers	-	-	-	5	101	7.2
Jimison, H.; Gorman. P.; Woods, S.; Nygren, P.; Walker, M.; Norris, S.; Hersh, W. [69]	10	Barriers and drivers of health information technology use for the elderly, chronically ill, and underserved	-	-	-	6	90	9.0
Haddon, L. [39]	18	Social exclusion and information and communication technologies: Lessons from studies of single parents and the young elderly	-	-	-	7	89	4.9

R—rank; C—the total number of citations per year; C/Age—average citations received by years.

**Table 3:** Authors with the highest number of publications.

R.	Name	Affiliation	Country	Tfi	WoS						Scopus					
					fi	LA	SA	C	C/fi	h	fi	LA	SA	C	C/fi	h
1	Hanson, E.	Linnaeus University	Sweden	9	5	1	0	124	24.8	4	9	1	0	298	33.1	6
2	Magnusson, L.	Linnaeus University	Sweden	8	5	2	0	124	24.8	4	8	4	0	268	33.5	6
3	Georgiou, A.	Macquarie University	Australia	3	3	0	0	26	8.7	2	3	0	0	24	8.0	2
4	Haux, R.	Intern. Acad. of Health Sciences Informat	Germany	3	3	2	0	59	19.7	3	3	2	0	54	18.0	3
5	Marschollek, M.	Medizinische Hochschule Hannover	Germany	3	3	1	0	59	19.7	3	3	1	0	54	18.0	3
6	Olve, N.	Linköpings universitet,	Sweden	3	1	1	0	3	3.0	1	3	1	0	53	17.7	3
7	Steinhagen, E.	Freie Universität Berlin	Germany	3	3	0	0	59	3.0	3	3	0	0	54	18.0	3
8	Tariq, A.	Queensland University of Technology	Australia	3	3	1	0	26	8.7	2	3	1	0	24	8.0	2
9	Vimarlund, V,	Linköpings universitet,	Sweden	3	1	0	0	3	3.0	1	3	2	0	53	17.7	3
10	Warburton, J.	La Trobe University	Australia	3	3	1	0	14	4.7	2	3	1	0	18	6.0	2
11	Westbrook, J.	Macquarie University	Australia	3	3	0	0	26	8.7	0	3	0	0	24	8.0	2
12	Wolf, K.H.	Medizinische Hochschule Hannover	Germany	3	3	0	0	59	19.7	3	3	0	0	54	18.0	3
13	Wulf, V.	University of Siegen	Germany	3	3	0	0	1	0.3	1	3	0	0	9	3.0	2

R.—rank; Tfi—frequency (number of articles published); LA—Lead Author; SA—Second Author; C—the total number of citations received by the published articles; C/fi—average citations received by the published articles; h—Hirsch’s index.

**Table 4:** Main countries according to the affiliation of the authors.

R.	Country	WoS U Scopus			WoS				Scopus			
		Authors	Authorships	Centers	fi	hi%	C	h	fi	hi%	C	h
1	Germany	109	169	40	8	6.6%	81	4	9	5.6%	101	5
2	United States	88	94	48	21	17.4%	455	10	26	16.0%	617	13
3	Australia	47	59	21	17	14.0%	95	6	18	11.1%	131	8
4	United Kingdom	41	42	26	9	7.4%	71	5	26	16.0%	758	8
5	Spain	32	36	19	10	8.3%	116	4	12	7.4%	171	6
6	Sweden	29	51	16	9	7.4%	167	5	16	9.9%	369	8
7	Italy	29	32	11	6	5.0%	30	3	8	4.9%	38	3
8	Japan	27	28	13	3	2.5%	26	2	8	4.9%	52	4
9	France	22	23	13	5	4.1%	28	3	6	3.7%	43	3
10	Finland	21	26	12	7	5.8%	37	4	9	5.6%	58	5
11	Taiwan	17	18	9	4	3.3%	237	2	6	3.7%	37	4
12	Netherlands	12	14	6	7	5.8%	19	3	6	3.7%	28	3
13	Portugal	11	11	5	4	3.3%	39	3	5	3.1%	64	4

R.—rank; fi—frequency (number of articles published); hi%—relative frequency; C—the total number of citations received by the published articles; h—Hirsch’s index.

**Table 5:** Most productive Journals.

Journal Title	Tfi	%	WoS				Scopus			
			fi	C	h	Q	fi	C	h	Q
Educational Gerontology	11	6.4%	11	250	8	Q4	11	328	8	Q3
International Journal of Medical Informatics	6	3.5%	6	220	6	Q1	6	303	6	Q1
Health Informatics Journal	5	2.9%	1	3	1	Q3	5	80	5	Q2
Computers in Human Behavior	4	2.3%	4	64	3	Q1	4	83	3	Q1
Informatics for Health and Social Care	4	2.3%	3	32	2	Q4	4	45	3	Q3
Generations-Journal of the American Society on Aging	3	1.7%	3	9	2	Q4	1	8	1	Q4
Ageing and Society	3	1.7%	2	37	2	Q2	3	338	3	Q1
Journal of Medical Internet Research	3	1.7%	2	94	2	Q1	3	120	3	Q1
Studies in Health Technology and Informatics	3	1.7%	1	3	1	-	3	6	2	Q4
Technology and Disability	3	1.7%	1	1	1	-	3	105	2	Q4
Gerontechnology	3	1.7%	-	-	-	-	3	17	3	Q4

Tfi—frequency (number of articles published); C—the total number of citations received by the published articles; h—Hirsch’s index; Q—quartile.

**Table 6:** Main areas of knowledge.

WoS						Scopus					
Area	J	fi	C	C/fi	h	Area	J	fi	C	C/fi	h
Geriatrics and Gerontology	22	35	563	16.1	12	Medicine	48	82	2024	24.7	22
Health Care Sciences and Services	12	20	401	20.1	11	Social Sciences	42	58	1112	19.2	15
Computer Science	13	19	286	15.1	8	Computer Science	29	33	259	7.8	8
Education and Education Research	7	17	280	16.5	9	Nursing	22	29	548	18.9	12
Medical Informatics	9	17	388	22.8	10	Engineering	19	24	88	3.7	5
Engineering	9	10	45	4.5	3	Health Professions	11	16	87	5.4	5
Psychology	7	10	209	20.9	5	Psychology	10	15	652	43.5	7
Communication	7	8	41	5.1	4	Arts and Humanities	7	12	483	40.3	6
Nursing	5	7	88	12.6	3	Biochemistry, Genetics and Molecular Biology	9	10	113	11.3	4
Public, Environmental and Occupational Health	5	5	48	9.6	1	Business, Management and Accounting	4	4	30	7.5	2

J—journal; fi—frequency (number of articles published); C—the total number of citations received by the published articles; C/fi—average citations received by the published articles; h—Hirsch’s index.

## References

[1] United Nations (2017). World Population Ageing. Highlights. https://www.un.org/en/development/desa/population/publications/pdf/ageing/WPA2017_Highlights.pdf.

[2] Selwyn N., Gorard S., Furlong J., Madden L. (2003). Older adults’ use of information and communications technology in everyday life. Ageing Soc..

[3] Czaja S.J., Charness N., Fisk A.D., Hertzog C., Nair S.N., Rogers W.A., Sharit J. (2006). Factors predicting the use of technology: Findings from the center for research and education on aging and technology enhancement (CREATE). Psychol. Aging.

[4] Katz S. (2000). Busy bodies: Activity, aging and the management of everyday life. J. Aging Stud..

[5] Baltes P.B., Baltes M.M. (1990). Successful Aging.

[6] Lloyd-Sherlock P. (2000). Population ageing in developed and developing regions: Implications for health policy. Soc. Sci. Med..

[7] Gorman M. (2000). Development and the rights of older people. The Ageing and Development Report: Poverty, Independence and the World’s Older People.

[8] World Health Organization (WHO) (2002). Envejecimiento activo: Un marco político. Rev. Esp. Geriatr. Gerontol..

[9] Thompson A., Strickland A. (2004). Administración Estratégica.

[10] Czaja S.J., Lee C.C. (2007). The impact of aging on access to technology. Univers. Access Inf. Soc..

[11] Eastman J.K., Iyer R. (2005). The impact of cognitive age on internet use of the elderly: An introduction to the public policy implications. Int. J. Consum. Stud..

[12] Hudson R.B., Hudson R.B. (2005). Contemporary challenges to age based policy. The New Politics of Old Age Policy.

[13] Neves B.B., Amaro F., Fonseca J.R. (2013). Coming of (old) age in the digital age: ICT usage and non-usage among older adults. Soc. Res. Online.

[14] Rice R.E., Katz J.E. (2003). Comparing internet and mobile phone usage: Digital divides of usage, adoption, and dropouts. Telecommun. Policy.

[15] Marquié J.C., Jourdan-Boddaert L., Huet N. (2002). Do older adults underestimate their actual computer knowledge?. Behav. Inf. Technol..

[16] Morris A., Goodman J., Brading H. (2007). Internet use and non-use: Views of older users. Univers. Access Inf. Soc..

[17] Lobet-Maris C., Galand J.M. (2004). Seniors and ICT’s: A sense of Wisdom. Commun. Strategies.

[18] Neves B.B., Amaro F. (2012). Too old for technology? How the elderly of Lisbon use and perceive ICT. J. Community Inf..

[19] Charness N., Holley P. (2004). The new media and older adults: Usable and useful?. Am. Behav. Sci..

[20] McMellon C.A., Schiffman L.G. (2002). Cybersenior empowerment: How some older individuals are taking control of their lives. J. Appl. Gerontol..

[21] Morrell R.W., Mayhorn C.B., Bennett J. (2000). A survey of World Wide Web use in middle-aged and older adults. Hum. Factors.

[22] Wang L., Rau P.L.P., Salvendy G. (2011). Older adults’ acceptance of information technology. Educ. Gerontol..

[23] Mitzner T.L., Boron J.B., Fausset C.B., Adams A.E., Charness N., Czaja S.J., Dijkstra K., Fisk A., Rogers W., Sharit J. (2010). Older adults talk technology: Technology usage and attitudes. Comput. Hum. Behav..

[24] Czaja S.J., Sharit J. (1998). Age differences in attitudes toward computers. J. Gerontol..

[25] Charness N., Kelley C.L., Bosman E.A., Mottram M. (2001). Word-processing training and retraining: Effects of adult age, experience, and interface. Psychol. Aging.

[26] Pew Internet and American Life Project Generations Online in 2009. http://pewinternet.org/Reports/2009/Generations-Online-in-2009.

[27] Alvseike H., Brønnick K. (2012). Feasibility of the iPad as a hub for smart house technology in the elderly; effects of cognition, self-efficacy, and technology experience. J. Multidiscip. Healthcare.

[28] Vaportzis E., Giatsi Clausen M., Gow A.J. (2017). Older adults perceptions of technology and barriers to interacting with tablet computers: A focus group study. Front. Psychol..

[29] Ma Q., Chen K., Chan A.H.S., Teh P.L. (2015). Acceptance of ICTs by older adults: A review of recent studies. International Conference on Human Aspects of IT for the Aged Population.

[30] Renaud K., van Biljon J. (2008). Predicting Technology Acceptance and Adoption by the Elderly: A Qualitative Study. Proceedings of the 2008 Annual Research Conference of the South African Institute of Computer Scientists and Information Technologists on It Research in Developing Countries: Riding the Wave of Technology (Saicsit ‘08).

[31] Davis F.D., Bagozzi R.P., Warshaw P.R. (1989). User acceptance of computer technology: A comparison of two theoretical models. Manage. Sci..

[32] Demiris G., Thompson H., Boquet J., Le T., Chaudhuri S., Chung J. (2013). Older adults’ acceptance of a community-based telehealth wellness system. Inf. Health Soc. Care.

[33] Chen K., Chan A.H.S. (2014). Gerontechnology acceptance by elderly Hong Kong Chinese: A senior technology acceptance model (STAM). Ergon..

[34] Smith T. (2008). Senior Citizens and E-commerce Websites: The Role of Perceived Usefulness, Perceived Ease of Use, and Web Site Usability. Inf. Sci. Int. J. Emerg. Transdiscipl..

[35] Cotten S.R., Anderson W.A., McCullough B.M. (2013). Impact of internet use on loneliness and contact with others among older adults: Cross-sectional analysis. J. Med. Int. Res..

[36] Cotten S.R., Ford G., Ford S., Hale T.M. (2012). Internet use and depression among older adults. Comput. Hum. Behav..

[37] Wright K. (2000). Computer-mediated social support, older adults, and coping. J. Commun..

[38] Aroldi P., Colombo F., Carlo S. (2015). New Elders, Old Divides: ICTs, Inequalities and Well Being amongst Young Elderly Italians. [Nuevos mayores, viejas brechas: TIC, desigualdad y bienestar en la tercera edad en Italia]. Comunicar.

[39] Haddon L. (2000). Social exclusion and information and communication technologies: Lessons from studies of single parents and the young elderly. New Media Soc..

[40] Koop C.E. (1995). A personal role in health care reform. Am. J. Public Health.

[41] Fischer S.H., David D., Crotty B.H., Dierks M., Safran C. (2014). Acceptance and use of health information technology by community-dwelling elders. Int. J. Med. Inf..

[42] Hoenig H., Taylor D.H., Sloan F.A. (2003). Does assistive technology substitute for personal assistance among the disabled elderly?. Am. J. Public Health.

[43] Steele R., Lo A., Secombe C., Wong Y.K. (2009). Elderly persons’ perception and acceptance of using wireless sensor networks to assist healthcare. Int. J. Med. Inf..

[44] Kang H.G., Mahoney D.F., Hoenig H., Hirth V.A., Bonato P., Hajjar I. (2010). Center for integration of medicine and innovative technology working group on advanced approaches to physiologic monitoring for the aged. In situ monitoring of health in older adults: Technologies and issues. J. Am. Geriatr. Soc..

[45] Zwijsen S.A., Niemeijer A.R., Hertogh C.M. (2011). Ethics of using assistive technology in the care for community-dwelling elderly people: An overview of the literature. Aging Mental Health.

[46] Weiner M., Callahan C., Tierney W., Overhage J., Mamlin B., Dexter P., McDonald C. (2003). Using information technology to improve the health care of older adults. Ann. Int. Med..

[47] Liao H., Tang M., Luo L., Li C., Chiclana F., Zeng X.J. (2018). A bibliometric analysis and visualization of medical big data research. Sustainability.

[48] Diem A., Wolter S.C. (2013). The use of bibliometrics to measure research performance in education sciences. Res. Higher Educ..

[49] Ismail S., Nason E., Marjanovic S., Grant J. (2012). Bibliometrics as a tool for supporting prospective R&D decision-making in the health sciences: Strengths, weaknesses and options for future development. Rand Health Q..

[50] Broadus R. (1987). Toward a definition of “bibliometrics”. Scientometrics.

[51] Benavides-Velasco C.A., Guzmán-Parra V., Quintana-García C. (2011). Evolución de la literatura sobre empresa familiar como disciplina científica. Cuad. Econ. Dir. Empresa.

[52] Rowley J., Slack F. (2004). Conducting a literature review. Manage. Res. News.

[53] Gasparyan A.Y., Ayvazyan L., Kitas G.D. (2013). Multidisciplinary bibliographic databases. J. Korean Med. Sci..

[54] Norris M., Oppenheim C. (2007). Comparing alternatives to the Web of Science for coverage of the social sciences’ literature. J. Infometrics.

[55] Álvarez-García J., Durán-Sánchez A., del Río-Rama M., García-Vélez D. (2018). Active ageing: Mapping of scientific coverage. Int. J. Environ. Res. Public Health.

[56] Meho L.I., Rogers Y. (2008). Citation counting, citation ranking, and h-index of human-computer interaction researchers: A comparison of Scopus and Web of Science. J. Am. Soc. Inf. Sci. Technol..

[57] Corral J.A., Canoves G. (2013). La investigación turística publicada en revistas turísticas y no turísticas: Análisis bibliométrico de la producción de las universidades catalanas. Cuad. Turismo.

[58] Price D.J.S. (1956). The exponential curve of science. Discovery.

[59] Merigó J.M., Mas-Tur A., Roig-Tierno N., Ribeiro-Soriano D. (2015). A bibliometric overview of the Journal of Business Research between 1973 and 2014. J. Bus. Res..

[60] Selwyn N. (2004). The information aged: A qualitative study of older adults’ use of information and communications technology. J. Aging Stud..

[61] Charness N., Boot W.R. (2009). Aging and information technology use: Potential and barriers. Curr. Dir. Psychol. Sci..

[62] Heart T., Kalderon E. (2013). Older adults: Are they ready to adopt health-related ICT?. Int. J. Med. Inf..

[63] White J., Weatherall A. (2000). A grounded theory analysis of older adults and information technology. Educ. Gerontol..

[64] Hernández-Encuentra E., Pousada M., Gómez-Zúñiga B. (2009). ICT and older people: Beyond usability. Educ. Gerontol..

[65] Choi N. (2011). Relationship between health service use and health information technology use among older adults: Analysis of the US national health interview survey. J. Med. Internet Res..

[66] Vroman K.G., Arthanat S., Lysack C. (2015). “Who over 65 is online?” Older adults’ dispositions toward information communication technology. Comput. Hum. Behav..

[67] Torp S., Hanson E., Hauge S., Ulstein I., Magnusson L. (2008). A pilot study of how information and communication technology may contribute to health promotion among elderly spousal carers in Norway. Health Soc. Care Community.

[68] Magnusson L., Hanson E., Borg M. (2004). A literature review study of information and communication technology as a support for frail older people living at home and their family carers. Technol. Disability.

[69] Jimison H., Gorman P., Woods S., Nygren P., Walker M., Norris S., Hersh W. (2008). Barriers and drivers of health information technology use for the elderly, chronically ill, and underserved. Evid. Rep. Technol. Assess..

[70] Gluck M.A. (1990). Review of journal coverage overlap with an extension to the definition of overlap. J. Am. Soc. Inf. Sci..

[71] Bearman T.C., Kunberger W.A. (1977). A Study of Coverage Overlap Among Fourteen Major Science and Technology Abstracting and Indexing Services.

[72] Meyer D.E., Mehlman D.W., Reeves E.S., Origoni R.B., Evans D., Sellers D.W. (1983). Comparison study of overlap among 21 scientific databases in searching pesticide information. Online Rev..

[73] Lotka A.J. (1926). The frequency distribution of scientific productivity. J. Wash. Acad. Sci..

[74] Haux R., Hein A., Eichelberg M., Appell J.E., Appelrath H.J., Bartsch C., Buschermöhle M. (2010). The Lower Saxony research network design of environments for ageing: Towards interdisciplinary research on information and communication technologies in ageing societies. Inf. Health Soc. Care.

[75] Haux R., Hein A., Kolb G., Künemund H., Eichelberg M., Appell J.E., Bente P. (2014). Information and communication technologies for promoting and sustaining quality of life, health and self-sufficiency in ageing societies–outcomes of the lower Saxony research network design of environments for ageing (GAL). Inf. Health Soc. Care.

[76] Bradford S.C. (1934). Sources of information on specific subjects. Engineering.

